# A profile of French clergymen who sexually assaulted victims and a review

**DOI:** 10.1080/19585969.2024.2429453

**Published:** 2024-11-22

**Authors:** Bénédicte Aubet, Julia Marie, Philippe Portier, Florence Thibaut

**Affiliations:** a Clinical Psychologist Mcs, Ecole de Psychologues Praticiens, Paris, France; b Directeur d’études à l’Ecole Pratique des Hautes Etudes (PSL), Ecole Pratique des Hautes Etudes, Paris, France; c Department of Psychiatry University Hospital Cochin, University Paris Cité, INSERM U1266 Institute of Psychiatry and Neurosciences, Paris, France

**Keywords:** Catholic Church, priests, sexual abuse, sex offence, sex offenders, victims

## Abstract

Sexual abuse within the Catholic Church is a distressing concern. As part of the investigation conducted by the French Independent Commission on Sexual Abuse in the Church, we analysed available files of convicted sexual abusers between 1950 and 2020. We analysed the socio-demographic and clinical characteristics of French clergymen sex offenders. Thirty-five clergymen were included. Sexual assaults were documented for 176 individuals in total, including 153 minors (79.7% male) and 23 adults (52.2% female). No sex offender assaulted both juvenile and adult victims. Homosexuality was declared in 50% of the perpetrators. A past history of child sexual abuse was observed in 30% (*N* = 9) of juvenile offenders. The mean number of victims per offender was around 5, with the highest mean number in male or both sex juvenile offenders. More than 90% of the victims were known to the perpetrator. Both hands-on and hands-off sexual offences occurred in over 80% of male juvenile offenders compared to less than 6% of female juvenile offenders and less than 17% of adult offenders. Sex offenders within the Catholic Church present some specificities in comparison to non religious sex offenders, such as a higher number of male juvenile victims somewhat older and more often known. Personal, interpersonal and systemic factors, some of which being specific, interact to foster sexual offence. Prevention of sexual abuse within the Catholic Church is crucial.

## 1. Introduction

The issue of sexual abuse within the Catholic Church has been a significant and distressing concern. This phenomenon has deep roots and, has only recently been brought to the attention of the general public by victims and relayed through the media. Since then, numerous investigations have been conducted around the world and have revealed the extent of sexual abuse (Australia (1980–2015); John Jay College of Criminal Justice 2004; UK (The Roman Catholic Church 2020), The Netherlands (1945–2008), Ireland (Catholic institutions 1936–1989), Belgium (1960–2011; Chambre des représentants de Belgique 2011), Germany, France and more recently Portugal and Spain) (for review Terry 2015).

In Australia, the Royal Commission calculated, based on victim testimonies, that 7% of the clergy in office between 1950 and 2010 was accused of sexual abuse against minors (Royal Commission into Institutional Responses to Child Sexual Abuse 2017). The prevalence of sexual abusers among clergymen in office in Germany between 1946 and 2014 was estimated between 4.17 and 4.4% (Dressing et al. 2021 – archives of the Catholic Church; Frings et al. 2022). In the USA, 4.8% of clergymen in office between 1950 and 2002 have been credibly or substantiated accused of having committed juvenile sexual abuse (mostly in the 1960s and 1970s – archives of the Catholic church) (John Jay College of Criminal Justice 2004; Plante and McChesney 2011; Plante 2020) compared to 6% of teachers at public schools (a review by Shakeshaft and Cohan 1995). In France, Portier et al. (2021) estimated that between 2.9 and 3.2% of French clergymen were sexual abusers. Considering that approximately 50% of juvenile sex offenders have paedophilic **disorder** and that the prevalence of paedophilia has been estimated in the general male population at between 0.3 and 3.8% in Europe (Tenbergen et al. 2015; Plante 2020), the known prevalence of paedophilic disorders among clergymen, therefore appears comparable to what is declared in the general population.

The number of victims is probably greatly underestimated if we refer to what has been described in the general population (McGlone 2001; Drury et al. 2020; Bajos et al. 2021). Thus, a survey conducted by Bajos in 2020 (CIASE report), 1.16% of people who have practiced activities related to the Catholic Church between 1950 and 2020 (including schooling in a religious institution) reported any kind of sexual abuse (1.7% boys and 0.61% girls). This prevalence was higher than the one observed in public schools (0.34%) or sports clubs (0.28%); in the general population the lifetime prevalence of sexual abuse was 19.2% in women and 7.9% in men. In the Netherlands, a survey conducted in the general population over-40s showed that, 1.7% of the general population (2.7% of men and 0.7% of women) had been assaulted before the age of 18 by someone connected with the Church (Langeland et al. 2015).

The aim of this study was to analyse the socio-demographic and clinical characteristics of a sample of clergymen sex offenders and their declared victims in France.

## 2. Material and methods

### 2.1. Source of the sample

This study was conducted as part of a large investigation conducted by the ‘Commission Indépendante sur les Abus Sexuels dans l’Eglise’ (CIASE 2021) (*French Independent Commission on Sexual Abuse in the Catholic Church)* whose final report was published in October 2021 (https://www.ciase.fr/medias/Ciase-Final-Report-5-october-2021-english-version.pdf). An archival and socio-historical research led by a team from the École pratique des Hautes Etudes (EPHE) coordinated by Philippe Portier was conducted. This research was based on five types of source material: (1) answers to a questionnaire sent to all bishops and major superiors of the institutions affiliated to the Conference of Brothers and Sisters of France (CORREF) concerning the content of their archives in relation to the CIASE’s study; (2) the archives of the Church of France, including centralised archives and those of 31 dioceses and 15 institutes including historical, current and ‘secret’ archives. Only two refusals were mentioned, one from a diocese and one from an institute; (3) public archives of the Ministry of Justice, the Ministry of the Interior and the Gendarmerie Nationale; (4) a questionnaire-led survey of forty-eight members of clergy and religious orders on the evolution of training methods to chastity; (5) all testimonies sent directly to the CIASE or those which were publicly available; (6) publicly available sources including public statistics and French press data bases. The random selection of files was conducted earlier by the CIASE (EPHE (Ph. Portier)), which chose a set of archives, including judicial records from those made available by the Catholic Church for content analysis. All information collected was anonymous, and the researchers had no access to information that could identify the dioceses, priests, or victims.

The definition of sexual abuse used by the CIASE was described in Supplementary Table S1.

Thus, thirty-five files including court records of clergymen were made available to us from a 71-year period (1950 to 2020). The information available in these files varied greatly between files. Psychiatric assessments were present in thirty files, psychological assessments in eighteen files, and criminal records in nine. All these files were analysed using the same analysis chart, which was prepared by J Marie and F Thibaut based on Dressing et al. (2021) previous analysis of clergymen sexual offences in Germany (see Supplementary Table S1). The French National Ethics Committee (Comité National Consultatif d’Ethique) gave its approval on May 23rd 2019.

### 2.2. Description of the sample

This sample included the court records of thirty-three priests, one deacon and one consecrated layman. All of them have been prosecuted for at least one case of sexual offence, either on an adult (>18 years old) or a juvenile (<18 years old). The offence was committed between 1950 and 2020.

Sex offenders were categorised into four groups: the group of offenders of juvenile victims included three sub-groups: offenders of male victims, offenders of female victims and offenders of both male and female victims. The fourth group was made up of adult offenders (due to the small number of victims no subgrouping was used).

Sexual offences were defined according to the ‘hands-on and hands-off’ classification method (see Thibaut et al. 2020 and described in Supplementary Table S1).

### 2.3. Statistical analysis

Data analyses were conducted using JASP (Jeffreys’ s Amazing Statistics Program) version 0.14.1 statistical software (https://jasp-stats.org). The analyses carried out consisted of descriptive statistics (mean + SD and %). Given the type of statistical variables measured and the asymmetry of the available data, no comparison tests could be performed.

## 3. Results

### 3.1. Socio-demographic and clinical characteristics, sexual activity and criminal records of sex offenders

All cases included in our study involved clergymen (*N* = 35) with a mean age of 46 (SD = 14). Of these, 30 clergymen offended juvenile victims: 18 (60%) offended only male victims; 3 (10%) only female victims, and 9 (30%) both male and female victims; 5 clergymen offended only adult victims: 4 (80%) only female victims and 1 (20%) only male victims. No participants offended both minors and adults.

The total number of victims was 176.

#### 3.1.1. Sexual orientation declared

Homosexuality was the most frequent sexual orientation reported (51.43% of the total sample). The percentage of clergymen considering themselves heterosexual and bisexual was 24.28% and 24.28% respectively. The breakdown of sexual orientation reported by group of sex offenders is shown in Table 1.

**Table 1.** Reported sexual orientation by type of sexual offence.

	Type of offenders (*N* = 35)				
Sexual orientation (N; %)	Juvenile offenders (*N* = 30)	Offenders of male juveniles (*N* = 18)	Offenders of female juveniles (*N* = 3)	Offenders of male and female juveniles (*N* = 9)	Adult offenders (*N* = 5)
Homosexual	17 (53.30%)	15 (83.33%)	0 (0%)	2 (22.22%)	1 (20%)
Heterosexual	4 (16.67%)	1 (5.56%)	3 (100%)	0 (0%)	4 (80%)
Bisexual	9 (30%)	2 (11.11%)	0 (0%)	7 (77%)	0 (0%)

N: number of cases declared; %: percentage of cases

#### 3.1.2. Sexual activity declared independently of sexual assaults

Thirteen (52%) of all clergymen have had sexual activity with a partner prior to the assault(s) (28% with a woman, 22% with a man and 2% with both) (*N* = 35, available and valid data (v):25) including 7 (50%) of the male juvenile molesters (*N* = 18, v:14) as compared to none of the female juvenile molesters and 3 of 4 adult offenders (*N* = 5, v:4).

Fifteen (94%) reported masturbation independently of sexual assaults (*N* = 35, v:16), especially among juvenile male offenders or juvenile offenders of both sex. Five had already used adult pornography (*N* = 35; v:9), including 2 juvenile male offenders and 3 juvenile offenders of both sex. Furthermore, 3 offenders have watched child pornography (*N* = 35; v:5), including 2 juvenile male offenders and 1 juvenile offender of both sex. Specifically, one of them also had a history of other sexual abuse and a criminal record, but the other two did not have any history of sexual or criminal abuse.

In fact, one male juvenile offender and two juvenile offenders of both sex declared paedophilic fantasies, and in all three cases, a history of paraphilia was reported (2 had a criminal record of child abuse).

In addition, 8 (89%) of the clergymen declared they felt sexually frustrated (*N* = 35; v:9); seven (77.77%) of whom were offenders of juvenile male victims or of both sex. Insufficient data on paraphilic sexual fantasies or behaviours did not permit statistical analysis.

#### 3.1.3. Age at first sexual assault against a victim declared by the perpetrator

In juvenile offenders, the mean age at first sexual assault was 35.79 years (SD = 13.34) (*N* = 30; v:14) and even 33.38 years (SD = 12) in those who assaulted only male juveniles (*N* = 18; v:8); whereas adult offenders had a mean age at the time of their first assault of 65.5 years (SD = 0.70) (*N* = 5; v:2).

#### 3.1.4. Past history of childhood abuse in sex offenders

Twelve (36.36%) cases of past history of childhood abuse were reported in the total sample of sex offenders (*N* = 35; v:33). Of the 12 (36.36%) clergymen who had suffered abuse in their youth (*N* = 35; v:33), nine (34.6%) of them had specifically been sexually assaulted, all being juvenile offenders (*N* = 35; v:26). In this latter group, four were male juvenile offenders, 2 female juvenile offenders and 3 juvenile offenders of both males and females. More specifically, 4 (44.44%) were victims of fondling, 2 (22.22) of forced masturbation by their perpetrator, 1 (11.11%) of oral penetration, 1 (11.11%) was forced to masturbate the perpetrator and 1 (11.11%) was a victim of unspecified sexual abuse. Finally, a reoccurrence of the sexual abuse by the same perpetrator was reported in 7 (77.77%) of the cases. In 8 cases the abuse was perpetrated by a man, and only one sex offender was abused by both a boy and a girl. The mean age at their first sexual assault was 11 years old (SD: 2.92; median 10.5; *N* = 12; v:8). Only one of their perpetrators was convicted for the committed crime.

Physical violence and/or humiliation in childhood was reported in two male juvenile offenders and one adult offender (without sexual abuse in this latter case).

#### 3.1.5. Psychiatric past history

A psychiatric disorder was reported in one case (20%) of adult offenders (*N* = 5; v:5) as compared to 16 (55.17%) among juvenile offenders (*N* = 30; v:29); 24.2% of sexual offenders had psychoactive substance use disorders, mainly alcohol use disorders (18.2%) and all of them were juvenile offenders. The case of offender with an anxiety disorder was a juvenile offender (*N* = 30; v:28), as was the only case who reported paraphilia (2.8%) (*N* = 35; v:34), the 3 cases (8.82%) of suicidal behaviours (*N* = 35; v:34) and the 4 cases (11.80%) (*N* = 35; v:34) of depressive disorders (only observed in male juvenile offenders).

Finally, none of the subjects reported any history of eating disorder, obsessive-compulsive disorder, bipolar disorder, psychotic disorder according to the files. No systematic assessment was made for personality disorders.

#### 3.1.6. Somatic past history

Twenty-one (63.63%) offenders had a somatic history (*N* = 35 v:33); 20 (95.23%) of them were juvenile perpetrators and one case (4.77%) was an adult perpetrator.

Among the 20 juvenile molesters who had a somatic history, the nature of the disorder was specified in 5 cases: 3 cases of past history of neurological disorders (2 cases of meningitis, 1 case of epilepsy); one case of current Parkinson’s disease and one case of diabetes. Finally, the adult offender who reported a somatic history, had had prostatectomy in the past. The nature of the somatic disorders of the 15 other participants who reported such history remained unspecified.

#### 3.1.7. Previous care received by the offender

In the past, none of them received previous medical treatment for paraphilic disorders, but 2 (*N* = 35; v:34) received medical treatment for another reason. The first one was a male juvenile offender and the second one an offender of juveniles of both sex.

Four sex offenders underwent psychotherapy after the sexual assault, which was of psychoanalytical obedience in all cases. The first one exclusively abused male juveniles and the second one exclusively assaulted female juveniles. The other two abused juveniles of both sex.

#### 3.1.8. Complaints and prison sentences

Sexual assaults led to a complaint in 36.8% of cases of juvenile molesters (*N* = 30; v:21) and in 80% of cases of adult molesters (*N* = 5; v:3). The average duration of the prison sentence was 3.88 years (SD = 3.25) for male juvenile offenders (*N* = 18; v:7) compared to 6.50 years (SD = 2.12) for female juvenile offenders (*N* = 3; v:2), and 4.48 years (SD = 4.98) for juvenile offenders of both sex (*N* = 9; v:7). Comparatively, the mean duration of the prison sentence was 12.44 years (SD = 11.06) for adult offenders (*N* = 5; v:3).

Regarding general criminal history, 2 juvenile offenders – against males and against both sex – had sexual criminal histories and these were only past sexual offences on juveniles (*N* = 35; v:33).

Indeed, no perpetrator had a history of non-sexual offences.

In 14 cases (including 13 juvenile sex offenders) where the information was available, all sex offenders continued to practice after their conviction. Three juvenile offenders were judged according to canon law but the conclusions were not available (Table 2).

**Table 2.** Socio-demographic and clinical characteristics of the victims.

	Total sample of victims	Female victims		Male victims		Mean age of the victims (at first offence) (SD)	Mean number of victims (SD) (**MINI-MAXI**^1^)
		(N)	(%)	(N)	(%)		
Total sample (*N* = 35)	176	43	24.4%	133	75.6%	14.26 (7.09)	5.03 (**SD:** 4.39)
Adult offenders (*N* = 5)	23	12	52.2%	11	47.8%	27.07 (11.74)	4.6 (**SD:** 3.78) (2–11)
Juvenile offenders (*N* = 30)	153	31	20.3%	122	79.7%	12.51 (3.73)	5.1 (**SD:** 4.54) (v:28) (1–22)
Male juvenile offenders (*N* = 18)	94					12.52 (4.33)	5.22 (**SD:** 5.47) (1–22)
Female juvenile offenders (*N* = 3)	8					13 (1.67)	2.6 (**SD:** 1.15) (2–4)
Juvenile offenders with both male and female victims (*N* = 9)	51					12.43 (2.77)	5.67 (**SD:** 2.83) (2–11)

N: number of analysable files; SD: Standard Deviation; v: number of files with data available

^1^ MINI: minimum number of victims by perpetrator in each group ; MAXI: maximum number of victims by perpetrator in each group.

### 3.2. Characteristics of the victims and of the offences

#### 3.2.1. Socio-demographic and clinical characteristics of the victims

The mean age of victims was similar for male and female juvenile victims (around puberty) (Table 2). The percentage of male victims was higher (79.7%) for juvenile sex offenders in contrast with adult sex offenders where the percentage of female victims was higher (52.2%) (Table 2). The mean number of victims was slightly higher for male and both sex juvenile offenders compared to adult offenders, and mostly to female juvenile offenders.

In our study, 8/30 (22.86%) juvenile offenders assaulted only one victim. When these offenders were excluded, the mean number of juvenile victims increased above 6 (*M* = 6.63; SD = 4.49). One priest declared 22 victims (only boys).

#### 3.2.2. Socio-demographic and clinical characteristics of the victims according to the past history of sexual abuse of the offender

Nine out of the offenders with a history of sexual abuse had sexually offended juveniles. None of those with a past history of sexual abuse assaulted an adult victim.

Those without a past history offended slightly more male sex victims but less victims of both sex (Table 3) and they were slightly younger.

**Table 3.** Comparison of the number and sex of victims of perpetrators with a history of childhood sexual abuse versus those without a history of childhood sexual abuse.

			Male	Female	Male and Female	Mean number of victims (SD)
Sex offenders (SO) with a history of sexual abuse (*N* = 9)	Juvenile Offender	SO (9)	4 (44.44%)	2 (22.22%)	3 (33.33%)	
		V (47)	20 (42.55%)	6 (12.76%)	21 (44.68%)	***M* = 5.2 (SD: 3.61) in total** ***M* = 5 (SD: 4.69) for male victims** ***M* = 3 (SD: 3.41) for female victims** ***M* = 12.2 (SD: 17.02) for both male and female victims**
	Adult offender	0				
Sex offenders without any history of sexual abuse (*N* = 25)	Juvenile Offender	SO (20)*	14 (66.66%)	NS	6 (28.57%)	
		V (106)	74 (69.81%)	2 (1.88%)	30 (28.3%)	***M* = 5.29 (SD: 4.93) in total** ***M* = 6 (SD: 6.03) for male victims** **NS for female victims** ***M* = 5.6 (SD: 3.13) for both**
	Adult Offender	SO (5)	1 (20%)	4 (80%)	0	
		V (23)	11 (47.83%)	12 (52.17%)	0	***M* = 4.6 (SD: 3.78) in total** **Insufficient data (v:1; mean age = 21) for male victims** ***M* = 3 (SD: 1.42) for female victims**
	Total	SO (34)*	19 (55.89%)	6 (17.64%)	9 (26.47%)	
		V (176)	105 (59.66%)	20 (11.36%)	51 (28.98%)	***M* = 5.03 (SD: 4.39)**

*N* = 35, v: number of files with data available: 34. V: victim.

* Missing data in one case.

#### 3.2.3. Characteristics of the sex offences according to the status (known/unknown) of the victim

The victim was known to the perpetrator in 141 (95.92%) cases of juvenile victims and in 14 (60.87%) cases in adult victims (Supplementary Table S2).

In know victims, the mean number of sexual assaults was 2.26 ± 2.23 (mean ± SD) and the mean duration of the assaults of the same victim was 3.24 ± 1.98 years.

### 3.3. Types of sexual offences

Both types of sexual offences are detailed in Supplementary Table S3 according to the type of sex offenders. The total number of victims analysed was 176.

Hands-off sexual offences have occurred in 84.21% of male juvenile offenders (*N* = 32), compared to 5.26% of female juvenile offenders (*N* = 2) and 10.53% of adult offenders (*N* = 4). Juvenile male offenders more often undress themselves in front of the victim (like adult offenders), watch pornography with the victims (4 cases) or perform sexual acts in their presence (11 cases), whereas juvenile female offenders undress their victims (like adult offenders).

Hands-on sexual offences have occurred in 79.54% of cases when male juvenile offenders where involved (*N* = 140), compared to 3.41% when female juvenile offenders (*N* = 6) were involved and 17.04% in case of adult offenders (*N* = 30). Male juvenile offenders prefer touching above or beneath clothing, fondling of the offender’s genitals by the victim (20 cases) or of the victims’ genitals (55 cases), digital (2 cases), oral (7 cases) and/or anal (10 cases) penetration of the offender and/or the victim, anal penetration with an object (1 case). Female juvenile offenders prefer touching beneath clothing, fondling of the victim’s genitals (2 cases) and mostly digital penetration of the victim (3 cases). Adult offenders prefer touching above or beneath clothing, kissing the mouth, fondling of the victims’ genitals (7 cases) and mostly vaginal penetration of the victim (2 cases).

If the offender was interested in juvenile victims of both sex, the sexual act was more intrusive.

### 3.4. Location of sexual offences

For both groups of offenders, the majority of offences took place at the priest’s home: in 45 cases (64.3%) of juvenile offenders and 9 cases (69.2%) of adult offenders. They took place at their office in 3 cases (4.3%) of juvenile offenders and 2 cases (15.4%) of adult offenders, and finally at the victim’s home in 5 cases (7.1%) of juvenile offenders and 2 cases (15.4%) of adult offenders. In 19 cases (27.1%), juvenile offenders assaulted their victims during children’s summer camps compared to none of adult offenders.

For male juvenile molesters, the offence took place at the priest’s home in 25 cases (56.8%) versus 2 cases (100%) for female juvenile molesters and 75% of cases when the juvenile victims were of both sex. For male juvenile molesters, the offence took place during summer camps in 15 cases (34.1%) or 4 cases (17%) when the juvenile victims were of both sex, as compared to neither case of female juvenile molesters.

No correlation was found between the age of the perpetrator and the location of aggression.

## 4. Discussion

The issue of sexual abuse within the Catholic Church has been a significant and distressing concern. Sexual offences have numerous potential consequences on health, personal and professional life of the victims, which can last lifetime (Thibaut 2015).

Among the 35 cases made available by the CIASE, all clergymen perpetrators were males. Whatever religious communities – either Catholic or Protestant – or social environments considered, sexual offenders were men in more than 95% of cases (Dressing et al. 2017; Thibaut et al. 2020).

The mean age of juvenile offenders at their first sexual assault was around 36 years (slightly lower in those who assaulted only male juveniles). As a comparison, the mean age was higher in Germany (Dressing et al. 2017) and in the USA (Piquero et al. 2008) (39 years). In a French cohort of non-religious sex offenders (personal communication), the mean age at first sexual assault was lower (29 years in child sex offenders). The mean age at first sexual assault in clergymen adult offenders was over 65 years in this study, which was more suggestive of disinhibition linked to ageing.

As observed in our sample, most studies of clergymen sex offenders reported an over-representation of male minor victims whereas 30% of juvenile assaulters offended both male and female victims. In contrast, adult offenders of our sample assaulted female victims in four cases and a male victim in one case. According to Firestone et al. (2009), McGlone (2001) and Holt and Massey (2013), the majority of clergymen who sexually abuse juveniles prefer boys (68–72%) and about 12% abuse victims of both sex. They made the assumption that the male over-representation of victims could be linked to a higher opportunity to meet boys in the Catholic Church. The percentage of male young victims was 75–80% among clergymen compared to 45% in other social environments (public school, youth institutions, etc.) with somewhat older victims in the Catholic Church (Dressing et al. 2017; The Independent Inquiry into Child Sexual Abuse in Germany 2020, https://www.aufarbeitungskommission.de/english/; Frings et al. 2022). In Belgium, the USA, Ireland, the Netherlands, Australia and Ireland the percentage of male victims varied between 62.8 and 81% (53% in Portugal 2023) (for review Portier et al. 2021). In a general population of sex offenders, the population of men sexually attracted to male minor victims varied between 7% (exclusive attraction for boys declared among 2,500 paedophiles) and 40% (Hall and Hall 2007). In Germany the percentage of male victims was higher in the Roman Catholic church as compared to the Protestant church (69.8 vs. 45.3 % respectively) (Portier et al. 2021).

In our sample, homosexuality was the most frequent sexual orientation reported. These data suggest that the choice of juvenile victims could have been linked to sexual orientation, rather than to the opportunity to meet a male or a female victim. Those who offended juvenile victims of both sex were mostly bisexual. Juvenile female offenders were all heterosexual. Of the 89 clergymen examined by Dressing et al. (2017), 53.9% reported a heterosexual orientation, 34.9% a homosexual orientation, and 6.7% a bisexual orientation. In our study only 20% of adult offenders declared homosexuality. By comparison, among Catholic priests who had not committed sexual assaults, declared homosexuality varies between 17 and 50% with 9% of bisexuality (The Kansas City Star 1999; Wills 2000; McGlone 2001; Cozzens 2022).

The mean number of victims per offender was on average five, with the highest mean number of victims per offender observed when victims were male or both sex juveniles, and the lowest when victims were only female juveniles. As a comparison, in our population of 350 non-religious French sex offenders, the mean number of victims was 2 per offender (between 1 and 30 but only 10 offenders assaulted more than 5 victims) (personal communication). According to Dressing et al. (2017), a clergyman offender abused 2.7 victims on average, with a mean of 2 assaults per victim. This figure could go up to 5.4 victims if they excluded those who have attacked only one victim as compared to 6.6 in our study. In the USA, 3.5% of priests have abused 26% of victims with more than 10 victims per offender (John Jay College of Criminal Justice 2004). Thus, a small percentage of priests is responsible for a large number of the convictions. Prevention must focus primarly on the detection and treatment of these particular cases at high risk of recidivism.

In our sample, the mean age of juvenile victims was almost the same when male and female victims were compared (between 12 and 13 years-old). Terry et al. (2008) reported an age between 11 and 14 years old in male victims. The Portuguese Commission reported a mean age of 11.7 years in girls compared to 10.5 in boys (2023).

According to Groth and Oliveri (1989), some sex offender priests report being equally attracted to juveniles or adults while others prefer juveniles exclusively. In contrast, in our study, there was no case of both minor and adult victim by the same offender.

In our study, the victim was known to the offender in 95.92% of juvenile victims and, 60.87% of adult victims. When the juvenile victim was known, sexual offences were more often repeated on the same victim and over a period of several years. Fogler et al. (2008) reported that 68.8% of victims in the Catholic Church were assaulted several times compared to 73.1% among Protestants. In Catholic clergymen sexual abusers, offenders had committed several abuses (57.2%), most often lasting more than a year (27.5% of cases) or even 10 years in 5.3% of cases (Terry et al. 2008; Dressing et al. 2017; Portugal 2023). In one third of cases, sexual abuses ended when the victim moved away or became able to disagree (Portugal 2023).

In juvenile victims, the aggression mainly took place at the sexual offender’s home or during a holiday camp, more rarely at the victim’s home. In adult offenders, the assault took place at the priest or at the victim’s home. Indeed, the places in which the sexual assaults occurred were diverse but essentially linked to the practice of religion (religious education, religious retreat and any place in which religious people interact with juveniles in the context of their work) (John Jay College of Criminal Justice 2004, Portugal 2023; Rassenhofer et al. 2015; McGraw et al. 2019; Frings et al. 2022).

In the case of juvenile victims, only one third of victims filed a complaint as compared to 80% of adult victims, especially since the 2000s. With regard to convictions, they were rare and less severe when the victims were juveniles. In the survey conducted in victims in the general population (Bajos et al. 2021), 21% of victims of priests filed a complaint (compared to 13% in the general population for victims of non-religious offenders). In Portugal, only 4.3% of victims filed a complaint (2023).

Half of our sample had previous sexual activity with an adult prior to the assault. Sipe (1999) found that only 2% of US Catholic clergymen were truly celibate, 20% tried hard to maintain celibacy, and about 80% of clergymen were not celibate. Gregoire (2003) also noted that 50% of religious celibates were involved in long or short-term relationships. Loftus and Camargo (1993) reported that 28% had engaged in a sexual relation with an adult woman whereas 8.4% reported sexual misconduct with a minor. In our study, juvenile male or both male and female offenders seemed more prone to sexual behaviour with a greater feeling of frustration compared to the offenders of female or adult victims. Markham and Mikail (2004) found that the majority of clergymen juvenile molesters felt very lonely and lack satisfying relationships with adults especially in intimate relationships. They also had low self-esteem and low self-confidence (Terry et al. 2008; Montana et al. 2012). Calkins et al. (2015) reported that clergymen were more comfortable in relationships with teenagers, had fewer friends among peers. Furthermore, Lothstein (1999) reported that a number of priests did not view sex with men or boys as violation of their vow of celibacy.

For Langevin et al. (2000), some priests enter the clergy to seek refuge from paedophilic sexual urges; when disinhibiting factors such as age or alcohol were associated with these fantasies, sexual assault could occur. In the absence of standardised assessment tools, it was difficult to assess the number and type of paraphilias/paraphilic disorders present in our population. According to the data collected, in 3/30 cases of juvenile offenders a diagnosis of paedophilic disorder could be evoked. Similarly, in a previous study of 100 non religious convicted sex offenders under care order, a prevalence of 10% of paedophilic and/or exhibitionist disorder was reported (Tesson et al. 2012). According to Sipe (1990, 1995), Tallon and Terry (2008), McGlone (2001) and Ackerman and Khan (2012), 0.2 to 2.7% of clergymen sex offenders could be classified as paedophiles (i.e., having a preferential sexual interest in prepubescent juveniles), an additional 1.1 to 10.8% considered as ‘ephebophiles’ (i.e., having a sexual interest for middle-aged to older adolescents) and 27.8% having episodic paedophilic fantasies. The paedophilic-interest group had a significantly later onset of deviant behaviour and was more likely to socialise with the victim’s family and commit the sex offences at victims’ home. Paedophilic-interest priests also had the longest duration of abusive behaviour, and their victims waited longer to report the abuse (Tallon and Terry 2008). In contrast, non paedophilic priests appear to abuse any child to whom they have access. In previous studies conducted in general populations of child sex offenders, about 50% had a paedophilic disorder (for review Thibaut et al. 2020).

As previously reported in Terry et al. (2011) and Holt and Massey (2013), in our sample, there was little history of mental disorders other than psychoactive substance use disorders (essentially alcohol abuse only observed in juvenile offenders). A survey commissioned by the Permanent Council of the French Bishops’ Conference in November 2020 on the physical and mental health of priests reported that alcohol abuse was reported in 40% of priests; 8% suffered from alcohol dependence; and 20% showed symptoms of depression. Alcohol, and, sometimes illicit substances, can be used to facilitate sexual assault (Falkenhain 1998; Bryant 1999; Plante 1999, 2003; Markham and Mikail 2004; Kaufman et al. 2006; Piquero et al. 2008). In contrast, half of 191 French non-religious juvenile molesters had a history of psychiatric disorder (essentially depression or anxiety) and half were using regularly alcohol (personal communication).

According to the offenders’ reports: considering hands-off sexual offences, they were far more frequent in male juvenile offenders compared to female juvenile offenders or adult offenders. Considering hands-on sexual offences, they were also far more frequent in male juvenile offenders compared to female juvenile offenders or adult offenders. If the offender was interested in juvenile victims of both sex, the sexual act was more intrusive. In Portugal (Report of the Portuguese commission 2023 on victims), the type of sexual abuse depended also on the sex of the victim (mostly anal sex, fondling of genitals and masturbation) in boys and sexual proposals in girls.

According to the testimonies of the victims, the types of abuse perpetrated by clergymen were slightly higher as follows: in one third of the cases, it was a rape whatever the type of penetration (half oral and half anal or vaginal). Sexual fondling was carried out in two-thirds of cases (Bajos et al. 2021, CIASE report). Dressing et al. (2017) reviewed the type of sexual assaults perpetrated by clergymen in the Catholic Church. They described them as follows: 82.9% were ‘hands-on’ with vaginal and/or anal penetration (17.2%), oral penetration by the victim (7.4%) or of the victim by the aggressor (11.7%) – 17.1% were ‘hands-off’ including giving instructions to the victim to perform a sexual activity in front of the aggressor (14.7%), filming or photographing intimate scenes in the presence of the victim (10%) and forcing the victim to watch pornography (8.3%). The John Jay College of Criminal Justice (2004), Rassenhofer et al. (2015) and the Belgian parlementary report concluded that in more than a third of the cases, oral sex or anal and/or vaginal penetration occurred; in 71% of cases the abuse was repeated on the same victim (McGraw et al. 2019). In other religious or non-religious institutions, vaginal and/or anal penetration was more common (64.4% vs. 17.2% in the Catholic Church) (Dressing et al. 2017). In recent years, oral sex, as well as forced visualisation of pornography and masturbation, have increased.

Thirty percent of juvenile sex offenders were victims of sexual abuse by a male perpetrator in their childhood. Interestingly, no history of sexual assault was observed among adult offenders in our study. Similarly, Jespersen et al. (2009) observed a significantly lower prevalence of sexual abuse history among sex offenders against adults compared to sex offenders against children (OR = 0.51, 95% CI = 0.35–0.74), whereas the opposite was found for physical abuse. Approximately 30 to 40% of non religious sex offenders (especially juvenile offenders) have a past history of childhood sexual abuse and, at least as much, of physical (39.5%) or emotional (59%) violence (Thibaut et al. 2020). These people need specific care in relation to their own history of sexual abuse. We attempted to examine the types of sexual offences committed according to whether or not the perpetrator had a childhood history of sexual abuse. Juvenile sex offenders with a past history of sexual abuse offended more often both sex victims and younger victims. They also committed more hands-on sexual offences as well as anal penetration of the victim. None of these offenders had received any specific care after their own sexual assault.

The omnipotence of the clergy thus establishes an unbalanced relationship with the parishioners that can facilitate a strong hold on the victim and the culture of secrecy and denial around sexual offences. Indeed, all sex offenders’ clergymen in our sample continued their religious activity after their crimes. According to Sipe (1999), the absence of a clear ethical code of conduct for members of the clergy may also have played a contributing role. Thus, a member of the clergy in difficulty with his sexuality and relations with his peers can then establish affective and sexualised relations with juveniles (Sipe 1999). The Canon Code is however quite explicit that sexual contact with a juvenile is unacceptable, but this has not been translated into a clear code of conduct that could become a reference document taught and discussed during training. Given the lack of explicit definitions of the relationship between clergy and parishioners, especially with juveniles, and the sense of loneliness expressed by many clergymen, they would thus be particularly vulnerable when seeking emotional support and, when they do not know what sexual abuse means (Glancy et al. 2021). In addition, a past history of childhood sexual abuse (sometimes by a priest), which has never or rarely been taken care of, may increase the risk of becoming a sex offender, especially when alcohol is used.

## 5. Limitations of the study

Several limitations should be taken into consideration when interpreting our results. Firstly, due to the specificity of the study population and the difficulty of access to these files, our sample size was small. Secondly, although we homogenised the data obtained from the court files and expertises, some data were missing, which limited the performance of statistical analyses beyond descriptive statistics. In the future, it may be relevant to conduct a prospective study including more participants on a more homogeneous corpus of files. In addition, certain data, such as psychiatric history (including a history of paraphilia) or, somatic history (in particular neurological), which could have favoured the sexual assault are difficult to analyse because no standardised scales were used. Finally, sex offences were probably under-declared as already reported by DeLisi (cited in Drury et al. 2020), Drury et al. (2020) and Scurich and Johns (2019), the ratio of self‐report to official victims in non religious sex offenders was 3.32 (i.e., 69–74% of offenders disclosed unofficial sexual contact with victims when polygraph sessions were used).

Nevertheless, using court files as well as psychiatric and psychological expertises when available, we were able to describe the sexual offences perpetrated and to better understand the types of sexual assault perpetrated by these clergymen in adults, male and female juveniles. The histories of childhood sexual abuse among offenders were also well documented, which allowed us to analyse the offenders according to the presence or absence of a personal history of sexual abuse.

## 6. Conclusion

Sex offenders within the Catholic Church present a certain number of specificities, such as a higher number of male juvenile victims, with a higher number of victims who are somewhat older and mostly known, compared to the general population of sex offenders (Perillo et al. 2017; for review Terry 2015). A higher socio-cultural level of sex offenders was also reported in previous studies (Haywood et al. 1996). According to Wolfe et al. (2006) and to Tishelman and Fontes (2017), clergymen would use an additional element compared to non-religious sexual aggressors to get victims to accept the sexual assault, which is spiritual manipulation, using God as a technique of emotional manipulation.

Personal, interpersonal and systemic factors, some of which are specific to religious environments, interact in very complex ways to favour sexual assault within the Catholic Church. Few offenders have a preferential paedophilic sexual orientation (John Jay College of Criminal Justice 2004). Homosexual orientation was more frequently observed among clergymen as compared to the general population. According to Terry et al. (2011) and Glancy et al. (2021) poor psychosexual development, intimacy deficits, stressful work experiences and easy access to minors may have a significant impact on victim choice. Indeed, in most cases, sexual assaults result more from a failing personal context in an environment that gives the offender a feeling of protection and total impunity. Until recently sexual abuse of minors was considered as human failure and sin rather than as a criminal act. At the institutional level, the particular hierarchical structure of the Catholic Church can give a member of the clergy an illusion of power over the parishioners. A lack of rigour in the process of recruiting clergy members, especially when vocations are scarce, can lead to the recruitment of at-risk clergymen (Terry et al. 2011). The absence or insufficiency of awareness of the risk of sexual assault and of the boundaries that should be established in emotional relationships with juveniles can increase the risk of sexual assault. This might be increased by the absence of supervision or tutoring. As clergymen are presumed incapable of immoral conduct, sexual assaults may thus be more easily concealed, which in turn may facilitate further offences (for review see also Dressing et al. 2017; Glancy et al. 2021).

Finally, it seems important that the Catholic Church should cooperate actively in research programs allowing a better understanding of these problems in order to prevent them. National anonymous epidemiological studies on the sexuality of members of the clergy, such as those already carried out in the general population, will be essential to improve the prevention of sexual abuse.

The establishment of a code of good practices to which members of the clergy in difficulty could refer is an important step forward (CIASE report). Priests should be trained from the seminary on the sexual and relational problems that can arise with parishioners in the context of role-playing for example, thus breaking their feelings of shame, loneliness and/or humiliation in case of relational and/or sexual difficulties. They should be encouraged to seek counselling and psychological help. A close supervision and support (mentoring) for any new member of the clergy admitted to the seminary seems essential. The American Conference of Bishops (2015) made a number of recommendations in 2015. The French CIASE, in its public report published in October 2021, made also a number of recommendations to the French Catholic Church (www.ciase.fr).

## Supplementary Material

Supplemental Material
