# Supplementary material for: A profile of French clergymen who sexually assaulted victims and a review
Source: Dialogues Clin Neurosci. 2024 Nov 22;26(1):77–88. doi: 10.1080/19585969.2024.2429453 (PMC11587733; doi:10.1080/19585969.2024.2429453)
Supplement: Supplemental Material [file TDCN_A_2429453_SM9555.zip › Supplementary Table 1.docx]

**Supplementary Table 1.**

**Analysis grid (J Marie and F Thibaut)**

Age of priest at time of first sexual assault

Age of priest at time of sexual assault on the victim

Sexual orientation 1(yes) / 0 (no)

Homosexual

Heterosexual

Bisexual

Past history of abuse 1(yes) / 0 (no)

Emotional

Physical

Sexual abuse

Past history of sexual abuse 1(yes) / 0 (no)

Fondling

Type of penetration

Masturbation

Encouragement to masturbate

Sexual molestation

Age of occurrence

Male perpetrator

Female perpetrator

Repetition of act 1(yes) / 0 (no)

Psychological care after the abuse 1(yes) / 0 (no)

Complaint 1(yes) / 0 (no)

Conviction of perpetrator 1(yes) / 0 (no)

Criminal record 1(yes) / 0 (no)

Sexual

Non-sexual

Psychiatric history 1 (yes)/0 (no)

Alcohol abuse

Addiction

Eating disorders

Anxiety disorder

Depressive disorder

OCD

Bipolar disorder

Psychotic Disorder

Personality disorder

Sexual disorder/History of paraphilias

Suicidal behavior

Treatments 1(yes) / 0 (no)

Paraphilic disorder

Other

Somatic history

Neurological (specify)

Endocrinological (specify)

Urological (specify)

Other (specify)

Head trauma

Age

Loss of consciousness 1(yes) / 0 (no)

Sexual activity 1(yes) / 0 (no)

Sexual intercourse before priesthood

Masturbation

Pornography use

Child pornography use

Frustration

Paraphilic fantasies 1(yes) / 0 (no)

Pedophilia

Exhibitionnism

Sadism

Masochism

Voyeurism

Paraphilic activity 1(yes) / 0 (no)

Judgment in canon law 1(yes) / 0 (no)

Continued to work as priest after the abuse 1(yes) / 0 (no)

Prison sentence 1(yes) / 0 (no) and duration of the sentence

Psychotherapeutic treatment 1(yes) / 0 (no)

Psychoanalytic

CBT

Supportive

Drug treatment

Type

Dosage

Duration

Efficacy

Side effects

Family history 1(yes) / 0 (no)

Psychiatric 1(yes) / 0 (no)

Paraphilias 1(yes) / 0 (no)

Sexual disorders (specify)

Parental addiction 1(yes) / 0 (no)

Father

Mother

Age of victim

Victim 1(yes) / 0 (no)

Boy

Girl

Both

Adult

Sexual assault committed

Number. of different victims

Number of sexual assaults per victim

Duration of sexual assaults per victim

Complaint 1(yes) / 0 (no)

Victim known to the perpetrator 1(yes) / 0 (no)

Rape

Oral penetration of victim

Oral penetration of perpetrator

Finger penetration of victim

Finger penetration of perpetrator

Vaginal penetration of victim

Anal penetration of victim

Anal penetration of offender

Penetration with an object

Sexual assault (type) 1(yes) / 0 (no)

Undressing of victim

Undressing of offender

Instructions to perform sexual activities in offender's presence

Film and/or photograph intimate moments

View pornography

Observe intimate moments

Ask sexually intimate questions

Perform sexual acts in the presence of the victim

Instructions to perform sexual acts in the presence of the offender

Instructions to perform sexual acts with other children/teenagers

Manipulation of offender's genitals

Handling the victim's genitals

Sexualized violence

Inappropriate touching above clothing

Inappropriate touching under clothing

Kissing on the mouth

Sado-masochistic abuse

Humiliating actions

Location of the abuse 1(yes) / 0 (no)

Priest's office

Priest's home

Victim's home

Summer camps

**Definition of sexual abuse (CIASE : https://www.ciase.fr/medias/Ciase-Final-Report-5-october-2021-english-version.pdf):**

The Commission decided to include within its scope: children; adults subject to protective supervisory measures as defined by civil law15; any person involved in a non-consensual sexual relationship in the context of a hierarchical relationship or relationship of spiritual guidance or power. With regard to the perpetrators of violence, the Commission included the following persons: bishops, priests and deacons of the diocesan clergy, members of men’s religious orders, whether priests or not, and sisters of congregations and institutes of consecrated life. This included trainee clerics and brothers (seminarians, novices and scholastics), members of “Associations of the Faithful leading a Communal Life”16, regardless of the diocese, religious institute or association to which they belonged, but not candidates – a stage prior to the seminary or novitiate - nor lay members of the Church, due principally to a lack of archives or statistics about these persons.

With regard to the acts in question, the Commission decided on a definition inspired by the Criminal Code but also sufficiently meaningful for the vast target audience of its appeal for testimonies: any sexual assault committed with or without violence, constraint, threat or surprise (rape, sexual assault other than rape, incest, exhibitionism, sexual harassment), any sexual exploitation (procurement) or any abuse of children (corruption of children, sexual propositions made to children, sexual abuse of children, the fixing, recording or transmitting of an image of a pornographic nature of a child).

**Description of hands-on and hands-off sexual offences**

Hands-off sexual offence excludes any form of physical contact with the victim (i.e., asking the victim to get undressed, the offender getting undressed itself, giving the victim instructions to perform sexual activities in the presence of the offender, filming and/or photographing intimate scenes **in presence of the victim**, **and forcing the victim to watch pornography**). Hands-on sexual offence includes any form of physical contact with the victim (i.e., touching above the clothing, touching beneath the clothing, oral penetration by the victim, oral penetration by the offender, vaginal and/or anal penetration) (Rosner, 2003).
