# Supplementary material for: A profile of French clergymen who sexually assaulted victims and a review
Source: Dialogues Clin Neurosci. 2024 Nov 22;26(1):77–88. doi: 10.1080/19585969.2024.2429453 (PMC11587733; doi:10.1080/19585969.2024.2429453)
Supplement: Supplemental Material [file TDCN_A_2429453_SM9555.zip › Supplementary Table 2.docx]

**Supplementary Table 2. Characteristics of sex offences according to the status (known/unknown) of the victims.**

|  | Total Number of victims (N=176) | Known victims | | | | Unknown victims | | | |
| --- | --- | --- | --- | --- | --- | --- | --- | --- | --- |
|  |  | N | % | Mean number of sexual offences (SD) | Mean duration of sexual offences per victim in years (SD) | N | % | Mean number of sexual offences (SD) | Mean duration of sexual offences per victim in years (SD) |
| Total number of victims | 176  (v:173) | 155 | 89.59  (155/173) | 2.22  (2.27) | 3.24  (1.98) | 18 | 10.40  (18/173) | 1.5  (1) | NS |
| Juvenile victims | 153  (v:147) | 141 | 95.92  (141/147) | 2.26 (2.23) | 3.24 (1.98) | 6 | 4.08  (6/147) | 2  (1.41) | NS |
| Male | 94 | 91 | 96.81  (91/94) | 1.68  (0.88) | 3  (1.81) | 3 | 3.19  (3/94) | NS | NS |
| Female | 8 | 8 | 100  (8/8) | 10  (0)  (2 cases) | 9  (0)  (1 case) | 0 | 0  (0/8) | **/** | **/** |
| Adult victims | 23 | 14 | 60.87  (14/23) | 1  (0) | NS  (NS) | 9 | 39.13  (9/23) | 1  (0) | Occurred once |

*Note*. N: number or percentage of analyzable data, NS: non specified, meaning that no data could

be analyzed; SD: Standard Deviation
