# Supplementary material for: A profile of French clergymen who sexually assaulted victims and a review
Source: Dialogues Clin Neurosci. 2024 Nov 22;26(1):77–88. doi: 10.1080/19585969.2024.2429453 (PMC11587733; doi:10.1080/19585969.2024.2429453)
Supplement: Supplemental Material [file TDCN_A_2429453_SM9555.zip › Supplementary Table 3 Abt.docx]

**Supplementary Table 3. Types of sexual offence by groups of sex offenders.**

| **Hands-on sexual offences** | Type of victims | Offences in total  (176) | Percent of offences |
| --- | --- | --- | --- |
| **Total** | Male juveniles | 140 | 79.54 |
|  | Female juveniles | 6 | 3.41 |
|  | Adult | 30 | 17.04 |
| **Touching above clothing** |  |  |  |
|  | Male juveniles | 10 | 7.14 |
|  | Female juveniles | 0 | 0 |
| Subtotal | Juveniles | 10 | 6.85 |
| Subtotal | Adults | 3 | 10 |
| **Touching beneath clothing** |  |  |  |
|  | Male juveniles | 20 | 14.28 |
|  | Female juveniles | 1 | 16.67 |
| Subtotal | Juveniles | 21 | 14.38 |
| Subtotal | Adults | 5 | 16.67 |
| **Kissing the mouth** |  |  |  |
|  | Male juveniles | 7 | 5 |
|  | Female juveniles | 0 | 0 |
| Subtotal | Juveniles | 7 | 4.79 |
| Subtotal | Adults | 8 | 26.67 |
| **Digital penetration of the victim** |  |  |  |
|  | Male juveniles | 2 | 1.43 |
|  | Female juveniles | 3 | 50 |
| Subtotal | Juveniles | 5 | 3.42 |
| Subtotal | Adults | 1 | 3.33 |
| **Digital penetration of the offender by the victim** |  |  |  |
|  | Male juveniles | 1 | 0.71 |
|  | Female juveniles | 0 | 0 |
| Subtotal | Juveniles | 1 | 0.68 |
| Subtotal | Adults | 0 | 0 |
| **Fondling of the victim’s genitals** |  |  |  |
|  | Male juveniles | 55 | 39.28 |
|  | Female juveniles | 2 | 33.33 |
| Subtotal | Juveniles | 57 | 39.04 |
| Subtotal | Adults | 7 | 23.33 |
| **Fondling of the offender’s genitals by the victim** |  |  |  |
|  | Male juveniles | 20 | 14.28 |
|  | Female juveniles | 0 | 0 |
| Subtotal | Juveniles | 20 | 13.69 |
| Subtotal | Adults | 1 | 3.33 |
| **Oral penetration of the victim by the offender** |  |  |  |
|  | Male juveniles | 6 | 4.28 |
|  | Female juveniles | 0 | 0 |
| Subtotal | Juveniles | 6 | 4.11 |
| Subtotal | Adults | 1 | 3.33 |
| **Oral penetration of the offender by the victim** |  |  |  |
|  | Male juveniles | 7 | 5 |
|  | Female juveniles | 0 | 0 |
| Subtotal | Juveniles | 7 | 4.79 |
| Subtotal | Adults | 1 | 3.33 |
| **Vaginal penetration of the victim** |  |  |  |
|  | Male juveniles | 0 | 0 |
|  | Female juveniles | 0 | 0 |
| Subtotal | Juveniles | 0 | 0 |
| Subtotal | Adults | 2 | 6.66 |
| **Anal penetration of the victim** |  |  |  |
|  | Male juveniles | 10 | 7.14 |
|  | Female juveniles | 0 | 0 |
| Subtotal | Juveniles | 10 | 6.85 |
| Subtotal | Adults | 1 | 3.33 |
| **Anal penetration of the offender by the victim** |  |  |  |
|  | Male juveniles | 1 | 0.71 |
|  | Female juveniles | 0 | 0 |
| Subtotal | Juveniles | 1 | 0.68 |
| Subtotal | Adults | 0 | 0 |
| **Anal penetration with an object** |  |  |  |
|  | Male juveniles | 1 | 0.71 |
|  | Female juveniles | 0 | 0 |
| Subtotal | Juveniles | 1 | 0.68 |
| Subtotal | Adults | 0 | 0 |

| **Hands-off sexual offences** | Type of victims | Offences in total (38) | Percent of offences |
| --- | --- | --- | --- |
| Total |  |  |  |
|  | Male victims | 32 | 84.21 |
|  | Female juveniles | 2 | 5.26 |
| Subtotal | Juveniles | 34 | 89.47 |
| Subtotal | Adults | 4 | 10.53 |
| **Fondling: Undressing of the victim** |  |  |  |
|  | Male juveniles | 10 | 31.25 |
|  | Female juveniles | 2 | 100 |
| Subtotal | Juveniles | 12 | 35.29 |
| Subtotal | Adults | 2 | 50 |
| **Undressing of the offender** |  |  |  |
|  | Male juveniles | 7 | 21.87 |
|  | Female juveniles | 0 | 0 |
| Subtotal | Juveniles | 7 | 20.59 |
| Subtotal | Adults | 2 | 50 |
| **Viewing pornography with the victim** |  |  |  |
|  | Male juveniles | 4 | 12.5 |
|  | Female juveniles | 0 | 0 |
| Subtotal | Juveniles | 4 | 11.76 |
| Subtotal | Adults | 0 | 0 |
| **Performing sexual acts in the presence of the victim** |  |  |  |
|  | Male juveniles | 11 | 34.37 |
|  | Female juveniles | 0 | 0 |
| Subtotal | Juveniles | 11 | 32.35 |
| Subtotal | Adults | 0 | 0 |
